# Emergency Responder Health: What Have We Learned from Past Disasters?

**DOI:** 10.1289/ehp.118-a346

**Published:** 2010-08

**Authors:** Bob Weinhold

**Affiliations:** **Bob Weinhold**, MA, has covered environmental health issues for numerous outlets since 1996. He is a member of the Society of Environmental Journalists

As the *Deepwater Horizon* disaster unfolds in the Gulf of Mexico, public health practitioners are having a sinking déjà vu feeling. Once again, environmental disaster has struck, and tens of thousands of emergency responders—some professionals, but many more volunteers—have swung into action, potentially risking their health as they work to clean up the worst oil spill in U.S. history. Veterans of similar disasters are wondering if historical lessons learned can help keep the damage to a bare minimum. But a paucity of hard data on emergency responder health makes it difficult even to ask the right questions.

“Emergency responders have not been adequately studied,” says Gina Solomon, codirector of the occupational and environmental medicine residency and fellowship program at the University of California, San Francisco, and a senior scientist with the Natural Resources Defense Council. “They tend to be ignored.”

Professional emergency responders such as firefighters may not put much emphasis on health effects studies. “They are going to do what they need to do regardless of their own safety,” says Don Donahue, executive director of the Center for Health Policy & Preparedness at the Potomac Institute for Policy Studies, who has firefighters in his extended family. “They have to be a little crazy by definition.”

That heroic approach is manna for the people they save, but firefighters may pay a price with disorders such as cancer. Studies of cancers in firefighters have had mixed results, but there is evidence linking this occupation with brain, thyroid, esophageal, bladder, testicular, prostate, and cervical cancers, as well as melanoma and Hodgkin disease (Bates^1^ and Ma et al.^2^ are two such studies).

Health risks tend to be even greater for the many nonprofessional emergency responders who rush to the scene of crises such as oil spills, terrorist attacks, hurricanes, train derailments, and chemical releases. Those workers often don’t have the training and advanced equipment that protect professional emergency responders to some degree. These and many other factors make it a daunting challenge to protect the health of emergency responders during disasters.

## Helping Others, Hurting Themselves

Two days after the 11 September 2001 collapse of the World Trade Center (WTC) in New York City, the U.S. Environmental Protection Agency (EPA) said in a press release that “[m]onitoring and sampling conducted on Tuesday and Wednesday have been very reassuring about potential exposure of rescue crews and the public to environmental contaminants. . . . The EPA and OSHA [the U.S Occupational Safety and Health Administration] will work closely with rescue and cleanup crews to minimize their potential exposure.”^3^

In hindsight we understand those were hollow assurances, and ongoing research is showing that problems sustained by WTC responders aren’t diminishing much in number or severity. Of the estimated 60,000 to 70,000 emergency responders,^4^ at least 6,500 suffered significant new or worsened respiratory symptoms,^5^ and after seven years, many workers still experienced abnormal lung function.^6^ More than 1,700 also suffered serious probable mental health issues, with some still affected up to five years later.^7^ Still others showed diminished chemosensory perception after three and a half years—a serious concern for these workers given that the ability to detect particular odors is a critical safety concern for emergency responders.^8^

About 10,000 of the responders—some of whom are simply anticipating possible future health problems—are included in the pending settlement of financial claims that totaled about $713 million as of 10 June 2010.^9^ The money could finally begin flowing by October, nine years after the disaster.

Oil spills also are a source of adverse health effects in emergency responders, although only 7 of the world’s 38 supertanker accidents have been studied for human health effects, according to a 2010 review by Francisco Aguilera et al.^10^ After the 2002 *Prestige* supertanker accident that damaged the northern coast of Spain and nearby areas of France, the longer people worked on the cleanup, the more health problems they had. Volunteers who were not briefed on the use of personal protective equipment were less likely to use such equipment and also had significantly more nausea, vomiting, dizziness, headaches, and throat and respiratory problems. Some disorders lasted more than 20 months. Workers also exhibited decreases in prolactin and cortisol plasma concentrations and elevated levels of metals in their blood. Nearly one-fifth of those who cleaned birds suffered injuries, and many workers in a variety of settings had significant DNA damage. But even among those who used personal protective equipment such as masks and special clothing, these measures were sometimes less beneficial than expected.

There is very little publicly available science on physical health effects among cleanup workers following the *Exxon Valdez* oil spill in 1989. However, on 1 July 2010 the U.S. House Committee on Energy and Commerce requested such information held by ExxonMobil, and the company is cooperating with the request, says committee spokeswoman Karen Lightfoot. Once the committee has reviewed the information, it hopes to share it with others in the near future, she says.

Since the explosion of BP’s *Deepwater Horizon* oil rig on 20 April 2010, a range of acute health problems have been reported as more than 40,100 emergency responders work to clean up the spilled oil. Toxics of concern include the oil itself (including a pervasive oil mist) and its constituents benzene, toluene, ethylbenzene, and xylene; gases and particulate matter from intentional oil burning; and the mixture of crude oil and dispersants used to help break down the oil in the water. The nearly unprecedented scale of the pollution and the lengthy periods of time responders are working have many people concerned about short- and long-term health effects in both responders and the general public.^11^–^13^ For acute problems alone, 967 workers had reported injuries or illnesses as of June 20—the latest figures from the National Institute for Occupational Safety and Health (NIOSH).^14^ Only seven cases have been attributed to exposures to oil or other chemicals, but it’s impossible to say how many of the accidents treated and incidences of dizziness, nausea, and fainting may have resulted from a reaction to toxic exposures, and neither NIOSH nor other independent medical personnel have verified incident data reported by BP.^14^

Relatively small disasters also are a source of risk for emergency responders. When a 1991 train derailment near Dunsmuir, California, plunged a tanker car into the Sacramento River, about 19,000 gallons of metam sodium spewed into the water. The reaction between the pesticide and the water quickly formed noxious substances including methyl isothiocyanate, hydrogen sulfide, and carbon disulfide. Among the emergency responders were local prisoners who spent considerable time in the water removing dead fish; about two-thirds suffered dermatitis on their feet and ankles. Other state and federal employees doing similar work had no dermatitis. Later analysis revealed that most of the unharmed people quickly changed their wet clothes when their work was done.^15^

## Substandard Protection?

Several studies have concluded that 11–16% of the general population is substantially more vulnerable to toxic exposures than the remaining population.^16^–^19^ Percentages of first responders harmed in the emergencies already mentioned tended to fall in or near this range; at least 10% of WTC emergency responders got sick, as did 19% of the people who cleaned oiled birds after the *Prestige* accident and 7% of the residents (including many emergency responders) after the 1993 *MV Braer* supertanker accident off the Shetland Islands.^10^ In some cases, the percentages were far higher—53% for area residents, including cleanup workers, following the 1999 *Erika* supertanker accident near Brittany, France,^10^ and 64% for the Dunsmuir prisoners.^15^

The rough correlation of these numbers isn’t a coincidence. “I am sure that first responders’ susceptibility to toxic exposures is on the same continuum as the general population’s,” says Lynn Goldman, a professor of environmental health sciences at the Johns Hopkins Bloomberg School of Public Health, who has studied the Dunsmuir incident. However, she cautions that the specific type of vulnerability varies from person to person and is unpredictable, and that professional responders, moreover, may be a little less vulnerable due to the “healthy worker” effect.^20^

Michael Crane, an assistant professor of preventive medicine at the Mount Sinai School of Medicine, agrees it’s impossible to predict who may get sick while working during an emergency. He says the science remains too poorly developed to use genetic information, biomarkers, or family history as predictive tools.

Nevertheless, there are still several options for gauging when emergency responders need to take added precautions. One is simply to rely on human senses. However, many toxics are difficult to smell at airborne concentrations that can harm health. Moreover, stopping—even briefly—to assess how a situation smells runs counter to the traditional responder mentality of instantly doing what he or she needs to do.

Another option is to quickly check postdisaster health symptoms in the general population—if the general population is being affected, even though folks are farther from the source of the exposure and aren’t laboring hard in proximity to the toxicants, that’s a warning signal for the workers. In the case of the *Deepwater Horizon* disaster, residents as far as 50 miles inland have complained about strong odors, says Marylee Orr, executive director of the Louisiana Environmental Action Network. Others have had to double their asthma medication, she says, and some have been barely able to breathe. However, at a June 22–23 Institute of Medicine workshop on health effects of the spill, Mary Currier, Mississippi’s state health officer, reported that “so far none of the surveillance data indicate increases in human illness that could relate back to oil or dispersant exposure.”^21^

Relying on either of these approaches would be consistent with the precautionary principle, which holds that it’s wise to assume a substance is dangerous until it’s proven safe. Linda Rae Murray, president-elect of the American Public Health Association and chief medical officer of the Cook County (Illinois) Department of Public Health, supports the precautionary principle. But she acknowledges that both having a quick emergency response and protecting the responders is very difficult.

In order to better quantify such decisions, the dominant current practice among public health officials is to compare sampled toxic emissions in a disaster area with available health benchmarks. Based on monitoring data for a dozen or so pollutants taken at a small number of airborne and land-based fixed and mobile monitors, the EPA consistently said during the first seven weeks of the *Deepwater Horizon* disaster there was no serious health threat, although the agency acknowledged there could be a pronounced smell similar to that of a gas station and that short-term problems such as headaches and nausea were possible.^22^

Relying on available health standards and limited monitoring concerns Murray: “Situations like that in the Gulf bring into clear focus a problem we usually ignore,” which is that OSHA standards are “horribly outdated,” and no standards accurately account for factors such as real-world exposure to multiple toxics. She also says there are no standards that address the exposures and stress endured during extra-long crisis workdays that can go on for weeks or months; “all our metrics go out the window,” she says. Her concerns about stress are supported by a growing body of science that shows psychological stress in conjunction with toxic exposures can increase adverse health effects.^23^–^26^ Crane also notes that the limited monitors don’t document exposures in the microenvironments of thousands of individual workers, and land-based monitoring doesn’t capture elevated levels that may exist offshore nearer the fresh oil.

Joseph Hughes, program director of the Worker Education and Training Program at the National Institute of Environmental Health Sciences (NIEHS), agrees there are serious problems with current standards for these kinds of situations. “There’s tremendous uncertainty in using these [pollution monitor] readings and being able to have some kind of accurate risk framework,” he says. He notes that the EPA has been working on this issue with its collaborative Acute Exposure Guideline Levels program, which has been operating since 1986, but much remains to be done before there can be a comprehensive set of standards that address these limitations.

## A High-Stakes Juggling Act

Even when emergency responders use the best available protection, many problems can still arise. For instance, Murray says, “Wearing a respirator is not benign”—longer-term use can tax the lungs, and a respirator, especially combined with a protective suit, can contribute to substantial heat stress. The *Deepwater Horizon* Unified Area Command—which includes BP and the U.S. Coast Guard (USCG), in consultation with OSHA—weighed the risks and benefits of using respirators during the first seven weeks of the *Deepwater Horizon* cleanup. BP spokesman Mark Proegler says that since no air standards were being violated, the company chose not to issue respirators to workers (under OSHA’s voluntary respirator requirements) and to reassign any workers who insisted on wearing a respirator. After consultation with NIOSH, BP has agreed to provide respirators to personnel involved in the *in situ* burning of oil, Proegler says.

According to Proegler, all such decisions are made mutually through the Unified Area Command, but many critics have questioned who is in charge of worker protection—or who should be in charge. However, in this emergency and others, laws that are based on factors such as the geographic location of the accident and the material involved dictate who is responsible for what, precluding some options.

Proegler says the Safety Unit of the Unified Area Command includes safety and industrial hygiene personnel from the USCG, BP, and OSHA. In the *Deepwater Horizon* response, although OSHA does not have jurisdiction offshore, the Unified Area Command has a written memorandum of understanding that defines a consultative role for OSHA. In addition, he says, the USCG and OSHA each have safety and industrial hygiene personnel at the Mobile and Houma command centers.

When considering the roles of public and private parties, Crane says, “There has to be a complementary role for both. It’s an important and necessary partnership, but it’s not always a happy marriage.” To diminish the impacts of any disagreements on emergency responders, he says it’s essential they hear the same guidance for protective practices from both the public and private parties because such mutual reinforcement is more likely to sink in.

Eight U.S. House committees worked in June and July on legislation that could change many aspects of emergency responses, potentially including supervision, protection, surveillance and research of health problems, and medical treatment. For instance, the proposed Oil Spill Accountability and Environmental Protection Act of 2010 that was being discussed in the House in July includes provisions requiring that the party responsible for an incident pay for personal injuries suffered due to that incident, says Lisette Morton, legislative director for Rep. Jerrold Nadler (D–NY). That would modify the Oil Pollution Act of 1990, which doesn’t stipulate such payments. However, Morton says it probably won’t be until at least September that the House and Senate may agree on a bill with these and other provisions.

Nadler says one overarching document that doesn’t need much revision at this time is the National Response Framework, a guide administered by the Department of Homeland Security that spells out how all parties should prepare for and respond to disasters. “[The framework is] good if it’s adhered to,” he says, but that didn’t happen for the *Deepwater Horizon* crisis, he adds. “Generally speaking, the government has significant authority it just didn’t fully utilize or adequately carry out,” he explains. “We’re not talking about a major overhaul, but rather fine-tuning and direction.”

The basic guidance in OSHA’s Hazardous Waste Operations and Emergency Response Standard (HAZWOPER),^27^ a primary guide for emergency responders, also helps create adequate protection if it’s followed, Hughes says. For instance, he notes that HAZWOPER generally adopts a precautionary approach, suggesting greater initial protection for workers and downgrading that only if incoming data confirm that lesser protection is warranted. “And that’s the approach we have consistently encouraged BP to take,” he says.

To better implement in the field what is on paper, Crane says it’s best to first inform volunteers they may be vulnerable to health effects from emergency response if they have existing health problems, then let them decide whether to work. However, economic considerations often mean workers feel they can’t afford to refuse hazardous work.

Once workers are in the field, Crane says it’s essential to follow the basics: educate them, train them for their specific task(s), identify the natural leaders, work within the culture of the group, explain and enforce job rules, closely monitor all workers, and make sure personal protective equipment is replaced or repaired if it becomes damaged.

When considering iffy situations such as the use of respirators in hot settings, Murray says one remedy is to use air-conditioned protective suits, although she acknowledges these are expensive and in short supply. Another option is to provide standard respirators and make sure workers take frequent breaks and are paid for their break times. She adds that supervisors and monitors need to be independent experts who aren’t obligated to please a company trying to save money.

## Up Next for the *Deepwater Horizon* Workers

Looking ahead for the *Deepwater Horizon* crisis, 40,119 emergency responders have voluntarily provided basic contact and *Deepwater Horizon* job description information to NIOSH as of July 16, allowing them to possibly be involved in postevent research efforts (although key baseline health data prior to exposure won’t be available), says NIOSH spokesman Fred Blosser. Some of that research is taking shape under the auspices of the NIEHS. The goal of the Gulf Long-Term Follow-Up (GuLF) Study is to evaluate more than 20,000 of the cleanup workers for a range of possible health effects, including respiratory, neurobehavioral, carcinogenic, immunologic, and mental health disorders, says Dale Sandler, chief of the Epidemiology Branch in the NIEHS Division of Intramural Research. Some of the study subjects would be workers who have enrolled in training but not yet been exposed, serving as a comparison group. The initial five years of the planned study would cost about $28 million, $8 million of which has already been allocated by the National Institutes of Health (BP officials did not respond to repeated inquiries about whether they would pay for this research or for any treatment that is needed). Sandler says up to 20 years of research is desirable in order to evaluate the potential for long-term effects such as cancer.

The research might begin as soon as September after the team, aided by NIEHS contractor SRA International, works with the agencies, organizations, and communities involved to finish designing the program, Sandler says. She explains that the study will not provide medical care or testing for participants, but participants will be able to inform their doctor about the overall study results—which are expected to be released as soon as various phases become available—and the doctor can proceed accordingly.

What health lessons will the *Deepwater Horizon* disaster yield for future emergency response workers—and will we heed them? Only time will tell. For today Crane hopes any lessons learned can help break the pattern seen in past disasters of unnecessary destruction and loss of life followed by lengthy periods of poorly controlled toxic exposures for emergency responders.

## Figures and Tables

**Figure:**
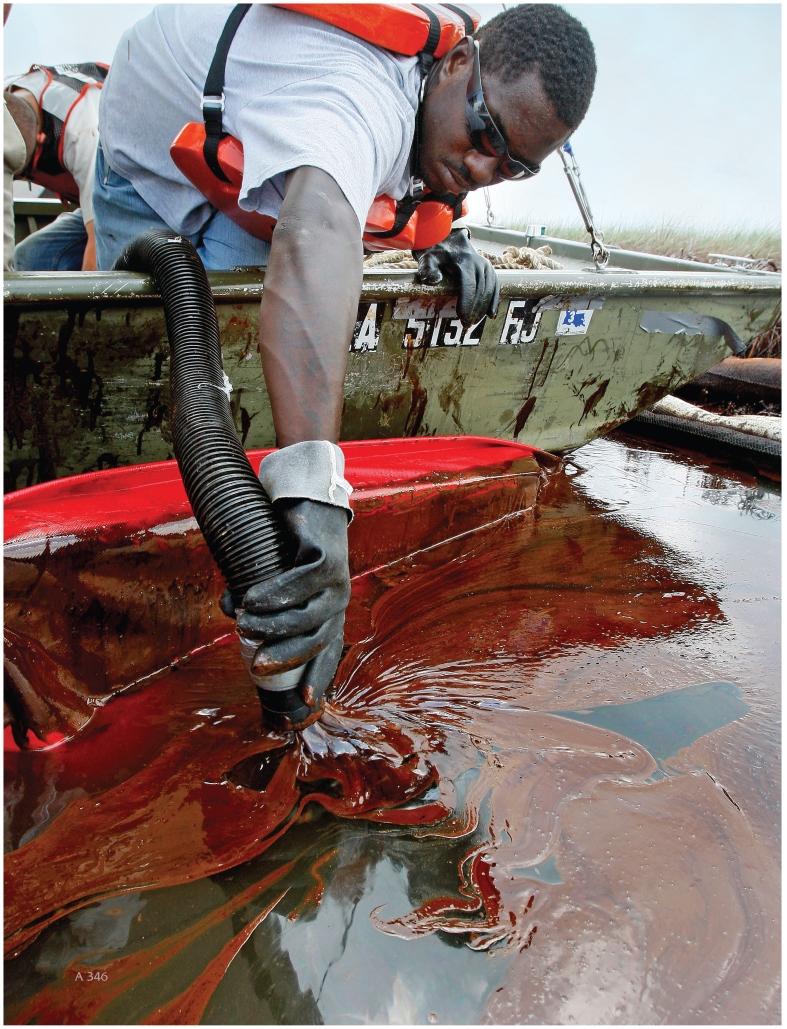


**Figure:**
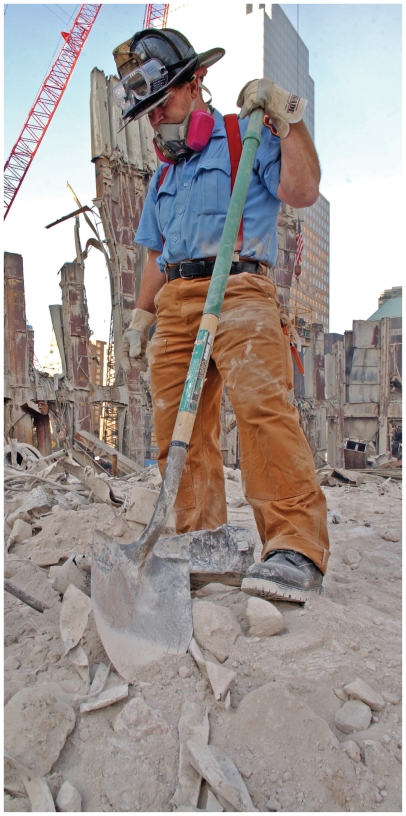
Will the Deepwater Horizon emergency response workers avoid the health fallout experienced by workers in past disasters? OPPOSITE: A cleanup worker vacuums surface oil in Barataria Bay, Louisiana, 20 June 2010; ABOVE: A firefighter pauses at WTC Ground Zero, 13 October 2001.

**Figure:**
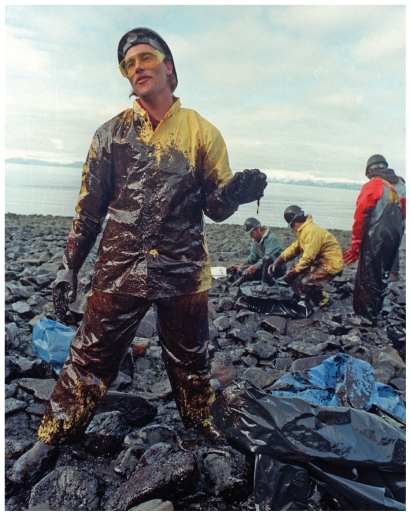
A volunteer cleans a beach after the Exxon Valdez spill, Prince William Sound, 13 April 1989. The Exxon Valdez spill presented many of the same worker health challenges as the Deepwater Horizon disaster but yielded little in the way of publicly available health data.
